# Advancing Cancer Research in Africa Through Early-Career Awards: The BIG Cat Initiative

**DOI:** 10.1200/JGO.18.00223

**Published:** 2019-04-22

**Authors:** Amanda L. Vogel, Jordan A. Freeman, Kalina Duncan, James Alaro, John J. Welch, Belmira Rodrigues, Verna Vanderpuye, Joe B. Harford, Makeda Williams

**Affiliations:** ^1^ Frederick National Laboratory for Cancer Research, Frederick, MD; ^2^ National Cancer Institute, Rockville, MD; ^3^ African Organization for Research and Training in Cancer, New York, NY; ^4^ Capacity Building for Innovation and Transformation, Potomac, MD; ^5^ National Institute of Mental Health, Bethesda, MD

## Abstract

**PURPOSE:**

The burden of cancer in Africa is growing rapidly, and increased cancer research on the continent is a critical component of an effective response. In 2010, the US National Cancer Institute, in partnership with the African Organization for Research and Training in Cancer, launched the Beginning Investigator Grant for Catalytic Research (BIG Cat) initiative to support cancer research projects conducted by early-career African investigators.

**METHODS:**

To date, BIG Cat has provided 18 awards of up to $50,000 to support 2-year cancer research projects. In 2017, the National Cancer Institute evaluated BIG Cat’s early outcomes for cancer research and impacts on career development and local cancer research capacity. Data collection consisted of a review of project documentation and a survey fielded to the 12 investigators who had completed their BIG Cat awards.

**RESULTS:**

BIG Cat–supported research projects have generated locally relevant findings that address a range of cancer sites and multiple areas of scientific interest. The 11 survey respondents produced 43 scholarly products (e.g., publications, presentations) about findings from their BIG Cat research. They reported increases in cancer research funding applications and awards after receipt of the BIG Cat award compared with before the award. They also reported increased resources for cancer research, participation in teaching and mentoring on cancer research, and supervision of cancer research staff. Investigators identified scientific mentoring as a key facilitator of the success of their BIG Cat projects and limited time and funding as key challenges.

**CONCLUSION:**

Findings provide early evidence that BIG Cat advanced locally relevant cancer research and facilitated career advancement and development of local cancer research capacity. Findings have implications for the design of future related efforts.

## INTRODUCTION

The burden of cancer in Africa is growing rapidly, and increased cancer research on the continent is a critical component of an effective response. In 2012, there were an estimated 846,961 new cancer cases and 591,169 cancer deaths in Africa.^1^ These numbers are expected to double in the next 20 years as a result of a confluence of factors, including population growth and aging, better control of infectious disease, increasing prevalence of cancer risk factors associated with economic transition, and growing population exposure to viruses linked to cancer risk.^2-4^

Cancer research conducted in Africa is necessary for the development and successful implementation of evidence-informed, locally appropriate approaches to cancer prevention and control.^2,5-7^ Yet in many African countries, cancer research capacity is inadequate because of multiple challenges, including competition for available funds in often meager health budgets; limitations in research infrastructure, workforce, and training; little foreign cancer research funding; and low policy prioritization of cancer research.^2,6,8^

To help to address the need for increased cancer research in Africa, in 2010, the National Cancer Institute (NCI) Center for Global Health (CGH), in partnership with the African Organization for Research and Training in Cancer (AORTIC), launched the Beginning Investigator Grant for Catalytic Research (BIG Cat), a pilot initiative that supports cancer research conducted by early-career African investigators.

Career development awards (e.g., the NCI Research Career Awards "K" Program^9^) are a key component of National Institutes of Health (NIH) activities to support the development of the next generation of health researchers. However, limited opportunities exist for foreign investigators to obtain these awards. Perhaps the best-known opportunities are from the NIH Fogarty International Center.^10^ BIG Cat addresses the need for early career support for cancer researchers on the African continent, specifically.

**CONTEXT SUMMARY**

**Key Objective** To support 2-year cancer research projects led by early-career African investigators to contribute to advancing cancer research conducted in Africa.
**Knowledge Generated** Funded research projects have addressed a range of cancer sites and areas of scientific interest and have been directly relevant to the African context, as indicated by translational applications. In addition to demonstrated scientific productivity (eg, publications), awardees have reported key indicators of cancer research career development, such as funding applications and awards, and increased local cancer research capacity, as indicated by increased human and material resources for future cancer research, for example. Receipt of research mentoring emerged as a key contributor to success.
**Relevance** The findings provide evidence to inform the design of future awards to build local capacity for cancer research.

The BIG Cat initiative has three goals: (1) to advance cancer research conducted on the African continent; (2) to support cancer research career development among African investigators; and (3) to build local capacity for cancer research in Africa. This article shares results from an early evaluation of BIG Cat and discusses implications for the design of future related efforts.

## METHODS

The BIG Cat initiative provides grants of up to $50,000 to early-career African researchers to conduct 2-year cancer research projects. Investigators must live on the African continent, and their research must be conducted in Africa and be directly relevant to Africa’s cancer burden. To date, BIG Cat has funded three cohorts (2010, 2013, and 2017) of six investigators each for a total of 18 awards. BIG Cat has been supported by NCI CGH in collaboration with the NCI Office of HIV and AIDS Malignancy (OHAM). Awards are administered and managed by AORTIC.^11^

In late 2017, CGH conducted an evaluation of the BIG Cat initiative. Its goals were to assess the early outcomes and impacts of BIG Cat on cancer research (eg, characteristics of supported research, dissemination of findings), cancer research career development (eg, subsequent grant applications and awards, increased professional focus on cancer research), and local cancer research capacity (eg, staffing, teaching, material resources) and to explore facilitating factors and challenges (eg, mentoring and resources for research).

Data collection consisted of a review of documentation from all 18 BIG Cat–awarded projects, with a focus on research project scope, goals, activities, and locations, and a self-administered 30-question Web-based survey fielded in fall 2017 to the 12 BIG Cat investigators in cohorts 1 and 2 who had completed their BIG Cat awards. Data collection was at 5 years and 2 years after completion of the award for cohorts 1 and 2, respectively. Investigators in cohort 3 were not included because their award period was ongoing. Eleven awardees completed the survey, including five from cohort 1 and six from cohort 2. Data were analyzed in Excel (Microsoft Corporation, Redmond, CA). The NIH Office of Human Subjects Research Protections approved this research.

## RESULTS

### Contributions of BIG Cat to Cancer Research

#### Breadth of science supported by the initiative.

The review of project documentation found that the 18 BIG Cat–supported research projects have contributed to the science on cancer prevention, etiology, early detection, diagnosis, treatment, prognosis, quality of care, and patient outcomes as well as cross-cutting issues (eg, sociocultural factors, health literacy) as applied to a diverse set of cancers within the African context (Table 1). Supported projects used research approaches and methods from a range of disciplines and fields, including genetics, surveillance and epidemiology, behavioral research, medicine, and health services research.

**TABLE 1:**
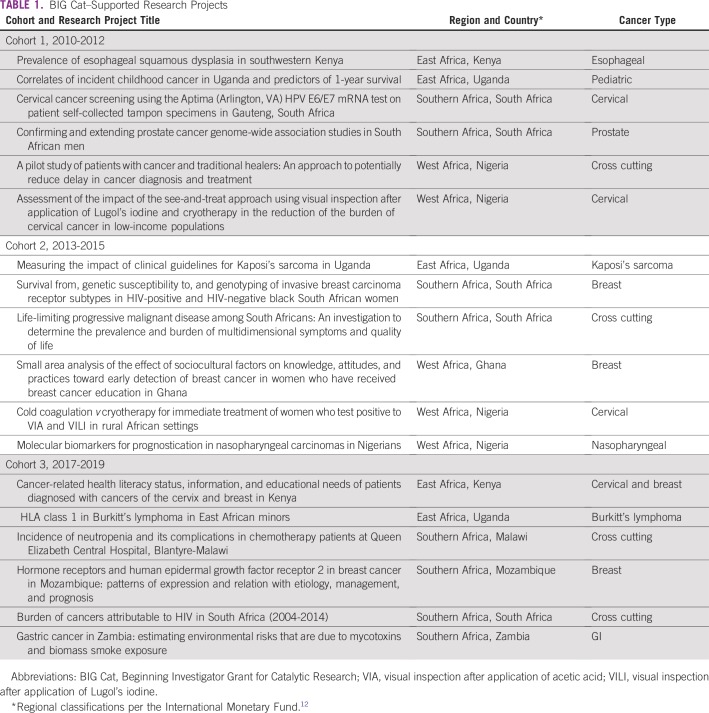
BIG Cat–Supported Research Projects

#### Local relevance of supported research.

Awardees have been located and their projects conducted in eight countries clustered in three regions of the African continent (East, West, and Southern Africa). Supported research projects have addressed research questions relevant to the African context. For example, projects have examined the impact of behavioral and sociocultural factors (eg, use of traditional healers, attitudes about early detection of breast cancer), tested portable and/or low-cost technologies suitable to low-resource settings (eg, cryotherapy and cold coagulation for cervical cancer, Lugol’s chromoendoscopy for esophageal cancer), and contributed to the evidence base on geographic variations in cancer prevalence.

#### Dissemination of findings.

**FIG 1:**
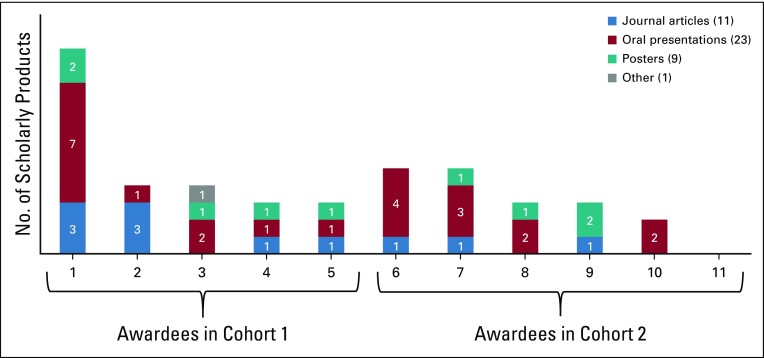
Scholarly products by respondent.

Of the 11 BIG Cat research projects represented in the survey data, by the time of the survey, 10 had produced a total of 43 scholarly products that featured findings from the BIG Cat–supported research project. These included 11 peer-review journal articles, as well as nine posters and 23 oral presentations at scientific conferences or meetings (Fig 1). For seven of the journal articles, the BIG Cat investigator was the first author. For one additional journal article, the BIG Cat investigator was the final author.

#### Translational applications.

Although not a requirement of the award, six of the 11 survey respondents, who were evenly distributed between the two cohorts, shared their BIG Cat project findings with a range of organizations, including nongovernmental organizations, community-based organizations, clinics/hospitals, and an academic institution. Respondents reported that these groups used BIG Cat project findings to help to inform development or enhancement of cancer prevention initiatives, design and/or implementation of cancer care improvement initiatives, and design of subsequent cancer research studies.

### Contributions of BIG Cat to Cancer Research Career Development

#### Subsequent cancer research grant applications and awards.

**FIG 2:**
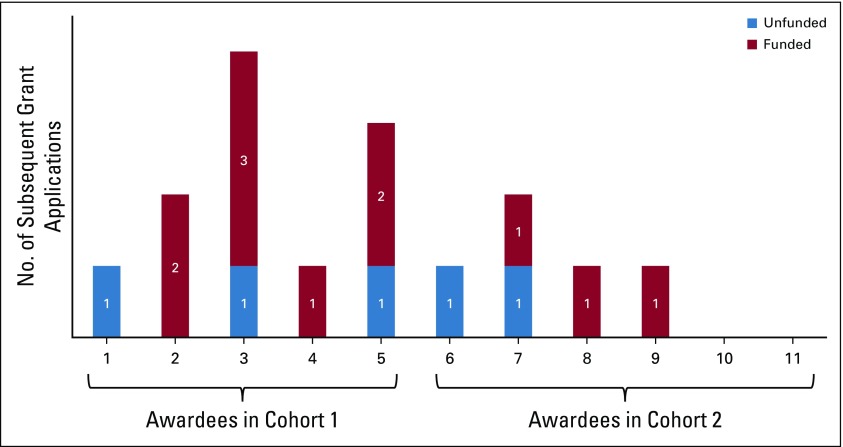
Subsequent funded and unfunded award applications by respondent and cohort.

Nine of the 11 respondents participated in applying for a total of 16 cancer research awards after receiving the BIG Cat award, of which 11 were funded (Fig 2). In comparison, four respondents had applied for a cancer research award before applying for BIG Cat, and two had received the award. When asked, “Did your BIG Cat project contribute in any way to your participation in this subsequent grant application/award?” respondents answered affirmatively for approximately three quarters of both grant applications (12 of 16) and awards (eight of 11). Respondents from cohort 1 were more likely to have applied for or received subsequent awards than respondents from cohort 2 (Fig 2). This finding reflects a temporal effect seen in many of the findings reported herein.

**FIG 3:**
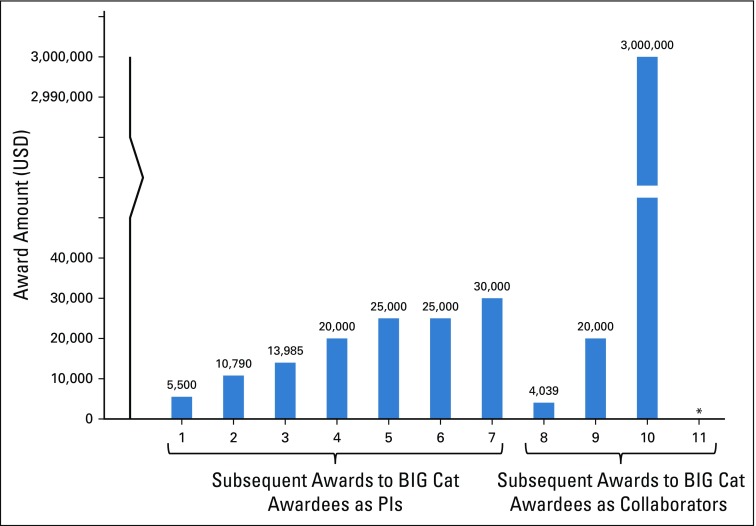
Dollar amounts of subsequent awards, by respondent role (principal investigator [PI] or collaborator). (*) Funded amount not reported. BIG Cat, Beginning Investigator Grant for Catalytic Research.

Respondents served as principal investigators on seven of the 11 subsequent cancer research awards, which ranged from $5,500 to $30,000 (Fig 3). They served as collaborators (eg, coinvestigator, subawardee) on the other four awards, which ranged from $4,300 to $3 million. The 11 subsequent awards were supported by a diverse group of 10 funders: the African Development Bank, the Burkitt Lymphoma Fund for Africa, the Conquer Cancer Foundation (two awards), the International Agency for Research on Cancer, the US NIH, the Pathological Society of Great Britain, the Tertiary Education Trust Fund of Nigeria, the Union for International Cancer Control, and two of the BIG Cat investigators’ universities.

#### Increased professional focus on cancer research as a result of BIG Cat.

Ten of the 11 respondents reported participating in one or more subsequent cancer research projects after receipt of the BIG Cat award, and all 10 reported that their BIG Cat projects contributed in some way to their participation in one or more of these subsequent projects. Most respondents (nine of 11) reported that participating in BIG Cat contributed to an increase in the amount of time they were able to dedicate to cancer research after their BIG Cat projects ended. In addition, most respondents (eight of 11) reported that participating in BIG Cat led to a change in professional title, position, or organization that allowed a greater professional focus on cancer research. All five respondents from cohort 1 and three from cohort 2 reported both of these outcomes, and one respondent from cohort 2 reported the latter of these outcomes.

### Contributions of BIG Cat to Local Research Capacity

Most respondents (nine of 11) reported that their participation in BIG Cat led to increased local cancer research capacity, as indicated by increased mentoring, staffing, time, and material resources for cancer research, and that this was sustained after their BIG Cat projects ended. Respondents from cohort 1 were more likely to report these outcomes (Table 2).

**TABLE 2:**
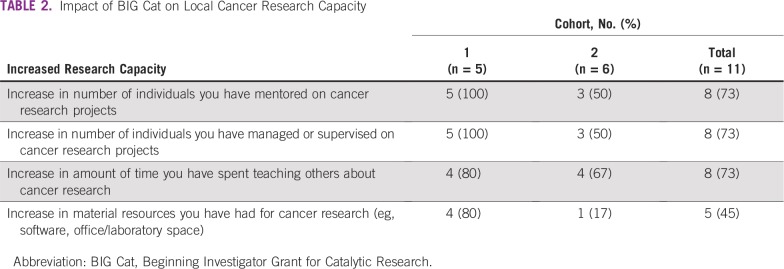
Impact of BIG Cat on Local Cancer Research Capacity

### Facilitating Factors and Challenges

#### Resources for BIG Cat research projects.

Nine of the 11 respondents reported that the BIG Cat award was their only source of funding for their BIG Cat projects. The other two respondents reported that the BIG Cat award provided more than half of the funding for their BIG Cat projects.

On a six-item scale of resources available for BIG Cat research projects, six of the 11 respondents had a score of five or six, four respondents had a score of three or four, and one respondent had a score of two (Table 3). There was no notable difference in response patterns by cohort except for one question on the duration of the award, which showed a dramatic difference between cohorts that could not be explained.

In text responses, respondents highlighted the importance of institutional research infrastructure (eg, statistical consulting service, institutional processes for research administration), skilled local research staff, and skilled local and international collaborators to the success of their BIG Cat projects, in addition to the resources identified in the scale.

**TABLE 3:**
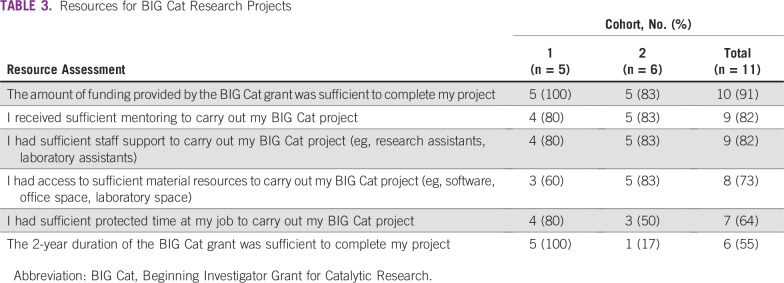
Resources for BIG Cat Research Projects

#### Mentoring.

Nine of 11 respondents reported having a research mentor for their BIG Cat projects, although this was not required by the award. Three mentors were located at the awardees’ institutions, and six were affiliated with a different institution in a different country. In text responses, respondents reported challenges communicating with distant mentors, that they effectively solved by using Internet-based videoconferencing. Respondents reported that mentors provided a range of benefits to their BIG Cat–supported research projects, including subject matter expertise and knowledge of local conditions and advising on research methods, project implementation, and development of publications and presentations. In addition, respondents described how mentors facilitated their professional development by helping them to hone research interests, create professional development plans, develop research networks, and apply for awards. Of note, all nine respondents who had mentors for their BIG Cat projects reported that these mentoring relationships continued after the completion of the projects. In addition, the two respondents who did not have mentors both identified insufficient research mentoring as a challenge to continued engagement in cancer research after their BIG Cat projects ended.

#### Limited funding and time.

When asked about a range of potential challenges to developing scholarly products about their BIG Cat projects (ie, journal articles, conference posters, and presentations), 10 of 11 respondents reported at least one challenge. There were two main reported challenges: a lack of funds to attend conferences/meetings to present BIG Cat project findings (six of 11 respondents) and insufficient time due to competing professional obligations and/or delays in the research project (six of 11 respondents). Only one respondent cited limited knowledge about how to write a research article or poster as a challenge.

When asked about a range of potential challenges to engaging in cancer research after their BIG Cat projects ended, 10 of 11 respondents reported at least one challenge. To echo the findings about challenges to developing scholarly products, the most commonly reported challenges to engaging in cancer research after BIG Cat were insufficient funding (six of 11 respondents) and insufficient time due to competing professional obligations (five of 11 respondents). A few respondents experienced other proposed challenges, including insufficient research mentoring (two of 11 respondents); insufficient staff support (two of 11 respondents); insufficient material resources for research, such as software and laboratory space (one of 11 respondents); and limited knowledge of the grant application process (one of 11 respondents).

Respondents from cohort 2 were more likely to report that lack of funding was a challenge both for developing scholarly products (five of six respondents from cohort 2 *v* one of five respondents from cohort 1) and for engaging in subsequent cancer research (four of six respondents from cohort 2 *v* two of five respondents from cohort 1). In contrast, respondents from the two cohorts were equally likely to report that insufficient time was a challenge both for developing scholarly products (three respondents in each cohort) and for engaging in subsequent cancer research (three of six respondents from cohort 2 and two of five respondents from cohort 1).

## DISCUSSION

An increase in cancer research conducted on the African continent is a critical component of an effective response to the rising burden of cancer in Africa. The BIG Cat initiative aims to advance cancer research conducted in Africa through a cancer research award for early-career African investigators. Findings from this early evaluation provide evidence that BIG Cat has been effective in supporting cancer research projects with direct relevance to the African context, facilitating career development among African cancer researchers, and building cancer research capacity in awardees’ local settings.

An emphasis of the BIG Cat award is to support research that is directly relevant to addressing the cancer burden in Africa. As reflected in Table 1, BIG Cat projects made contributions across scientific areas of interest and cancers, and all were highly tailored to their local settings. In addition, although translational applications were outside the scope of the BIG Cat award, more than half of survey respondents (six of 11) shared research findings with organizations that used the findings for translational applications, reflecting the local relevance of their research.

The increase in BIG Cat awardees’ cancer research funding applications (from four to 16) and awards (from two to 11) after receipt of the BIG Cat award is a signal indicator of career development. While this may reflect natural career progression over time, respondents attributed three quarters of subsequent funding applications and awards to their participation in BIG Cat, suggesting that BIG Cat contributed to career progression. Respondents’ mix of roles on subsequent awards (as principal investigators [64%] and collaborators [36%]) reflects development of both leadership and collaborative skills that are valuable for career success and scientific progress in this age of global research consortia and networks.^13^ In addition, five of the 11 reported journal articles included one or more US coauthors, and all of these articles included at least one coauthor from an NCI-Designated Cancer Center.

Awardees’ participation in teaching and mentoring on cancer research, supervision of cancer research staff, and increases in time and resources for cancer research continuing after their BIG Cat awards ended indicate that BIG Cat helped to build local cancer research capacity. Multiple awards incorporate mentoring, and BIG Cat awardees’ reports that mentoring was a key contributor to the success of their projects provide evidence for the value of this approach. Findings also suggest the value of mentoring regardless of the mentor’s geographic location. Respondents’ reports of Internet-based videoconferencing to support communication with distant mentors reflects the published evidence for the importance of face-to-face interactions to the success of scientific collaborations.^13,14^

Respondents experienced two main challenges—limited time and limited funding—that are commonly reported among investigators everywhere. However, low- and middle-income country (LMIC)–based investigators may encounter greater obstacles in both areas, such as fewer funding opportunities and greater demands on their time because of fewer clinical and research staff at their institutions compared with institutions in high-income countries.

BIG Cat demonstrates one approach to advancing cancer research in LMICs via awards to early-career investigators. A variety of additional approaches are needed to support the cancer research enterprise in these settings. In particular, initiatives that focus on institutional capacity building may address a challenge reflected in the geographic clustering of BIG Cat awardees—those regions that have the least cancer research, and therefore are in greatest need of research funding, may not have the capacity to generate competitive award applications. Ultimately, a combination of research and research capacity-building awards and increased institutional, national, and international investments in enhancing health systems are needed to advance cancer research most effectively in African countries and other LMICs where there is great need for increased cancer research.^3^

The findings should be interpreted in light of study limitations. One key limitation was that only 7 and 4 years had passed from receipt of BIG Cat awards for cohorts 1 and 2, respectively, to data collection. This was reflected in the findings. Compared with cohort 1, cohort 2 reported fewer subsequent grant applications and awards, more funding-related challenges, and fewer indicators of local research capacity development. These patterns likely reflect that additional time was needed for cohort 2 to achieve outcomes on par with cohort 1. It may be the case that this amount of follow-up time also was insufficient for cohort 1. Another key limitation was the lack of a comparison group, which would have helped to contextualize and interpret the findings. The self-reported nature of the data also should be considered.

The rising cancer burden on the African continent creates an imperative for increased local cancer research. The BIG Cat initiative supported locally conducted cancer research in Africa led by early-career African cancer researchers to advance cancer research on the continent. This evaluation provides early evidence for the potential benefits of such an award. Findings from this evaluation can help to inform the design of future efforts to support early-career LMIC investigators, pointing to the value of mentored research and the need to consider approaches to enable dissemination of findings and continued engagement in cancer research.
